# Multicolor Emissive Phosphorescent Iridium(III) Complexes Containing L-Alanine Ligands: Photophysical and Electrochemical Properties, DFT Calculations, and Selective Recognition of Cu(II) Ions

**DOI:** 10.3390/molecules27238506

**Published:** 2022-12-03

**Authors:** Xi Chu, Yichuan Huang, Wenhao Li, Shisheng Zhao, Hongyan Li, Aidang Lu

**Affiliations:** School of Chemical Engineering and Technology, Hebei University of Technology, Tianjin 300130, China

**Keywords:** Ir(III) complexes, L-alanine, sensing copper ions, fluorine-substituted cyclometalated ligands

## Abstract

Three novel Ir(III) complexes, (ppy)_2_Ir(L-alanine) (**Ir1**) (ppy = 2-phenylpyridine), (F_4_ppy)_2_Ir(L-alanine) (**Ir2**) (F_4_ppy = 2-(4-fluorophenyl)pyridine), and (F_2,4,5_ppy)_2_Ir(L-alanine) (**Ir3**) (F_2,4,5_ppy = 2-(2,4,5-trifluorophenyl)pyridine), based on simple L-alanine as ancillary ligands were synthesized and investigated. Due to the introduction of fluorine substituents on the cyclometalated ligands, complexes **Ir1**–**Ir3** exhibited yellow to sky-blue emissions (*λ*_em_ = 464–509 nm) in acetonitrile solution. The photoluminescence quantum yields (PLQYs) of **Ir1**–**Ir3** ranged from 0.48–0.69, of which **Ir3** with sky-blue luminescence had the highest PLQY of 0.69. The electrochemical study and density functional theory (DFT) calculations show that the highest occupied molecular orbital (HOMOs) energy of **Ir1**–**Ir3** are stabilized by the introduction of fluorine substituents on the cyclometalated ligands, while L-alanine ancillary ligand has little contribution to HOMOs and lowest unoccupied molecular orbitals (LUMOs). Moreover, **Ir1**–**Ir3** presented an excellent response to Cu^2+^ with a high selectivity, strong anti-interference ability, and short response time. Such a detection was based on significant phosphorescence quenching of their emissions, showing the potential application in chemosensors for Cu^2+^.

## 1. Introduction

Cu^2+^ participates in various biological processes in mammals, such as iron absorption, hematopoiesis, and metabolism [1,2]. Abnormal levels of Cu^2+^ can lead to acute hemolytic anemia and multiple neurodegenerative diseases [3,4]. Moreover, Cu^2+^ is also widely used in industry and agriculture, and the ecological environment could be damaged due to the emission of a large number of metal pollutants [5]. Thus, scientists are committed to developing accurate and dependable detective methods for Cu^2+^. Among various methods for the detection of Cu^2+^, fluorescence and phosphorescence detection methods based on changes in photophysical properties have received wide attention due to their high selectivity, rapid response, and simple operation [6,7,8]. Hence, the design and synthesis of novel luminescent materials to sense Cu^2+^ becomes a primary task.

Iridium complexes have gradually become the most promising phosphorescent materials due to their high PLQY (photoluminescence quantum yield) [9,10,11], easy modification of chemical structure [12,13], easy emission wavelength adjustment [14], and excellent chemical stability [15]. Thus, iridium complexes have been used as photosensitizers [16,17] and chemosensors for the detection of various analytes [18,19], showing excellent performance [20]. For example, Ir(III) complex **ZIr2** based on di(2-picolyl)amine (DPA) as a Cu^2+^ receptor was designed and synthesized, which exhibited phosphorescence quenching for addition of Cu^2+^ with high selectivity and good reversibility [8]. In another study, Ir(III) complex **Ir-L^14naph^** was designed with bidentate chelating pyrazolyl pyridine ligand as a copper-specific receptor, and the detection limit of Cu^2+^ was 20 nmol/L [21]. In these studies, the Cu^2+^ was recognized by combining with nitrogen heteroatoms in ancillary ligands of iridium complexes. However, the structure of ancillary ligands in the above complexes is complicated, which will increase the difficulty and cost of the synthetic process.

**Figure 1:**
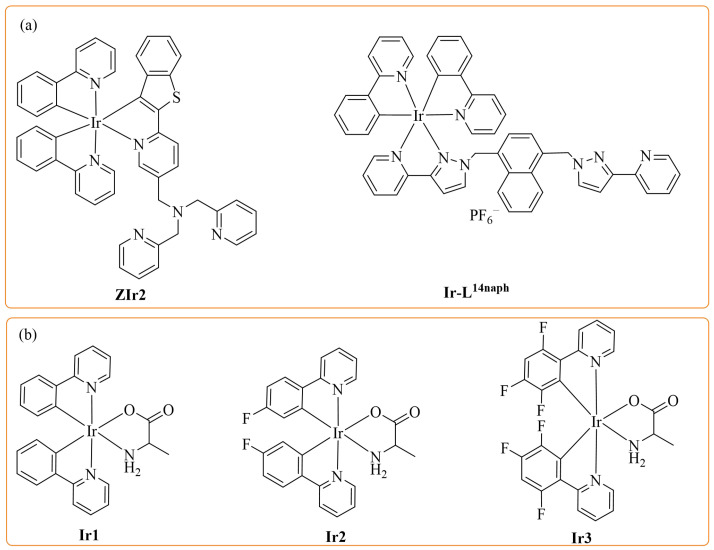
The structures of iridium complexes (**a**) described in the literature and (**b**) synthesized in this paper.

Amino acids, as cheap and easily available amphoteric substances containing oxygen and nitrogen atoms, can provide binding sites for sensing Cu^2+^. Hence, we selected the simple L-alanine as the ancillary ligand, which can simplify the synthesis method and reduce the material cost. Secondly, the introduction of C–F bonds into the cyclometalated ligands can reduce the rate of nonradiative process to enhance the PLQY and can increase the energy of the excited state to make the emission peaks shift to the blue region [22,23]. Herein, three novel Ir(III) complexes, (ppy)_2_Ir(L-alanine) (**Ir1**) (ppy = 2-phenylpyridine), (F_4_ppy)_2_Ir(L-alanine) (**Ir2**) (F_4_ppy = 2-(4-fluorophenyl)pyridine), and (F_2,4,5_ppy)_2_Ir(L-alanine) (**Ir3**) (F_2,4,5_ppy = 2-(2,4,5-trifluorophenyl)pyridine), were synthesized (Figure 1b). Complexes **Ir1**–**Ir3** presented yellow, green, and sky-blue emissions with high PLQYs up to 0.69. Furthermore, these complexes achieved as “turn-off” photoluminescence chemosensors of Cu^2+^ with high sensitivity.

## 2. Results and Discussion

### 2.1. Synthesis and Characterization

The cyclometalated ligands ppy and fluorine-substituted ligands F_4_ppy and F_2,4,5_ppy were prepared in high yields via the Suzuki coupling reaction. Complexes **Ir1**–**Ir3** were obtained by the Ir(III) chloro-bridged dimers ([(ppy)_2_Ir(μ-Cl)]_2_, [(F_4_ppy)_2_Ir(μ-Cl)]_2_, or [(F_2,4,5_ppy)_2_Ir(μ-Cl)]_2_), L-alanine, and potassium tert-butoxide in DMF under nitrogen atmosphere with moderate yields. Complexes **Ir1**–**Ir3** are air- and moisture-stable solids, and they are soluble in polar organic solvents including acetonitrile and dichloromethane. The structures of complexes **Ir1**–**Ir3** were confirmed by ^1^H, ^13^C, and ^19^F NMR spectroscopy (Figures S1–S8) and electrospray ionization mass spectroscopy (ESI-MS).

### 2.2. Photophysical Properties

**Figure 2:**
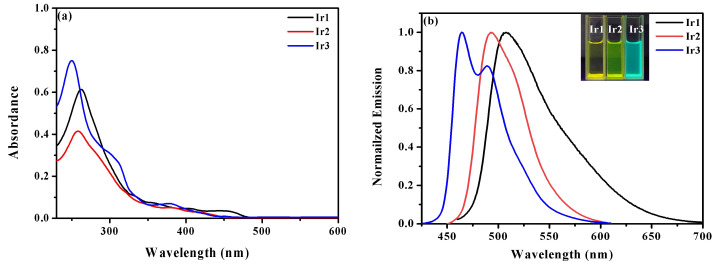
(**a**) UV-vis absorption and (**b**) normalized emission spectra of complexes **Ir1**–**Ir3** in acetonitrile solution (2.0 × 10^−5^ mol/L).

The UV-vis absorption spectra and normalized emission spectra of the complexes **Ir1**–**Ir3** in degassed acetonitrile (2 × 10^−5^ mol/L) are shown in Figure 2. The spectral data are summarized in Table 1. Under 320 nm, the absorption bands of complexes **Ir1**–**Ir3** with a high molar extinction coefficient in the level of 10^4^ L·mol^−1^·cm^−1^ can be attributed to the spin-allowed singlet ligand-centered (^1^LC) electronic transitions. The weaker bands in the 320–550 nm ranges are designated for metal-to-ligand (MLCT) and ligand-to-ligand (LLCT) charge transfer [24]. In 320–550 nm, the lowest energy absorption maxima of **Ir2** and **Ir3** are blue shifted in comparison with that of complex **Ir1**. The results indicate that the introduction of fluorine substituents to the cyclometalated ligands can widen the energy gaps of the absorption bands in the lowest energy region.

The maximum emission peaks of complexes **Ir1** and **Ir2** are 509 nm and 493 nm, and that of **Ir3** is 464 nm with the shoulder peak at 490 nm. The CIE color coordinates of **Ir1** and **Ir2** are (0.24, 0.67) and (0.14, 0.53) with yellow to green emission, those of **Ir3** are (0.13, 0.24) with sky-blue emissions. Compared with the emission peak of **Ir1**, a hypsochromic shift of 16 nm in **Ir2** and 45 nm in **Ir3** was observed, which can be attributed to the introduction of fluorine substituents in cyclometalated ligands. The lifetimes (τ) of complexes **Ir1**–**Ir3** measured at room temperature are 1.64, 1.67, and 1.46 μs, respectively, illustrating the phosphorescent character. The PLQYs of **Ir1**–**Ir3** in acetonitrile solution were measured to be 0.48–0.69, of which **Ir3** with F_2,4,5_ppy ligands had the highest PLQY of 0.69. The radiative (k_r_) and nonradiative decay rate constants (k_nr_) can be calculated through the equations k_r_ = PLQY/τ and k_nr_ = (1 – PLQY)/τ (Table 1). With the increase in the number of fluorine substituents in complexes **Ir2** and **Ir3**, the k_nr_ of **Ir2** and **Ir3** decreases and PLQYs of **Ir2** and **Ir3** increase. Therefore, the introduction of electron-withdrawing groups (-F) on the cyclometalated ligands can improve the energy of the MLCT state and reduce the rate of nonradiative process, which is beneficial to obtain iridium complexes with adjustable emission color and obtain phosphorescent materials with high PLQY.

### 2.3. Electrochemical Properties

**Figure 3:**
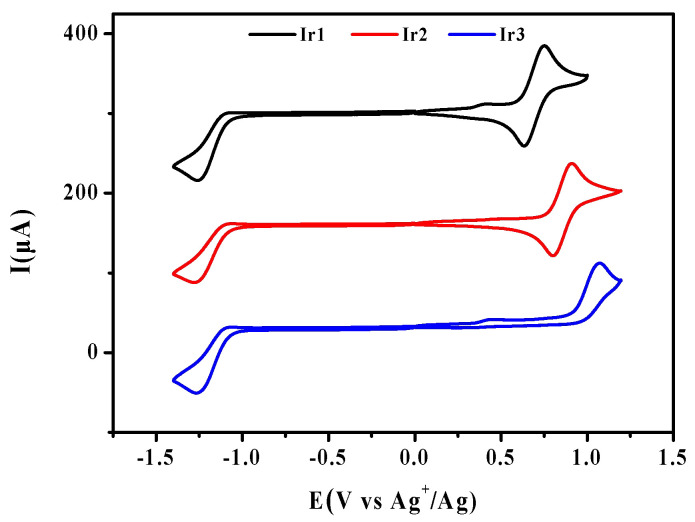
Cyclic voltammograms of complexes **Ir1**–**Ir3** in DCM: MeCN [1:1 (*v*/*v*)] solution.

The cyclic voltammograms of complexes **Ir1**–**Ir3** are presented in Figure 3, and Table 1 contains the data gathered. As shown in Figure 3, complexes **Ir1** and **Ir2** both have a pair of reversible oxidation waves between 0.50 and 1.20 V (vs Ag^+^/Ag), which are attributed to the oxidation process of Ir^3+^/Ir^4+^ [25]. Complex **Ir3** with F_2,4,5_ppy as the cyclometalated ligands displays an irreversible oxidation wave, suggesting electrochemical stability decreases with the increasing number of fluorine substituents [26]. **Ir3** > **Ir2** > **Ir1** is the trend of the oxidation potentials for complexes **Ir1**–**Ir3**, while the order of the highest occupied molecular orbital (HOMO) energy is **Ir3** < **Ir2** < **Ir1**. The findings suggest that the introduction of electron-withdrawing groups (-F) onto the cyclometalated ligands can increase its oxidation potential, so as to stabilize the HOMO energy. The complexes **Ir1**–**Ir3** have similar reduction potentials, and the irreversible reduction peaks appear in −1.00~−1.10 V (vs Ag^+^/Ag). According to the DFT calculations, the lowest unoccupied molecular orbitals (LUMOs) of complexes **Ir1**–**Ir3** are localized mainly on the pyridyl moieties of the cyclometalated ligands (Table 2), so the introduction of fluorine substituents has little influence on the reduction potentials and LUMO energy of the complexes.

### 2.4. Theoretical Calculation

**Figure 4:**
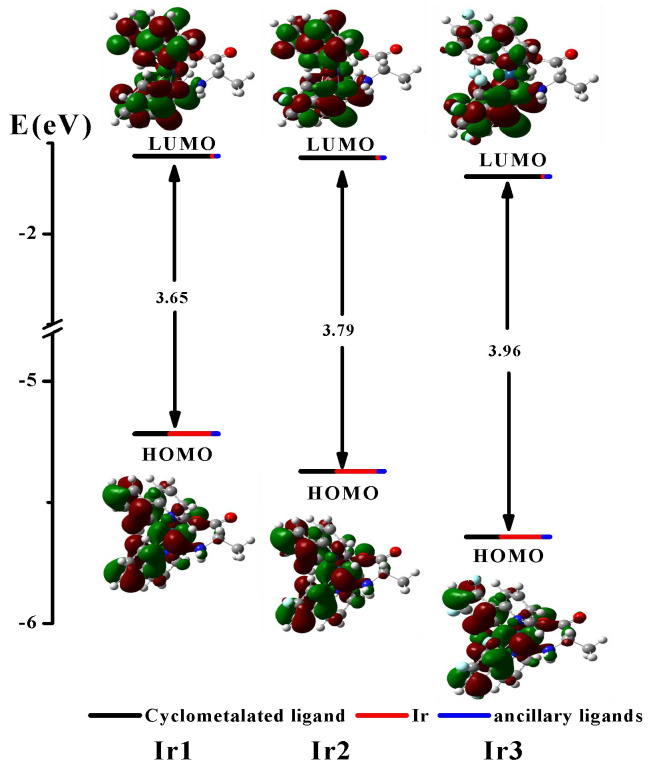
Frontier molecular orbital diagrams of complexes **Ir1**–**Ir3** were constructed using DFT, along with the percentage compositions of cyclometalated ligand (black line), iridium atom (red line), and ancillary ligand (blue line).

DFT and TD-DFT methods were used to explore the lowest-energy electronic transition of the complexes **Ir1**–**Ir3**. The energy and surface distributions of HOMOs and LUMOs for complexes **Ir1**–**Ir3** are presented in Figure 4 and Table 2, and Tables S4–S6 provide a summary of the calculated spin-allowed electronic transitions electron density distributions. The HOMOs of complexes **Ir1**–**Ir3** are predominantly located on the phenyl moieties (35.88–38.30%) of cyclometalated ligands and *d* orbitals of the iridium atom (42.12–43.80%). The LUMOs are mostly distributed over the π* orbitals of the pyridyl moieties of the cyclometalated ligand (66.01–67.02%) and have a small distribution on the iridium atom (4.49–4.79%). Thus, the introduction of substituents onto the phenyl moieties of cyclometalated ligands significantly affects the HOMO energy of the iridium complexes. The HOMO energy of complexes **Ir2** (−5.37 eV) and **Ir3** (−5.64 eV) is lower than that of **Ir1** (−5.22 eV), which can be attributed to the addition of fluorine substituents of iridium complexes. It can be proved that the introduction of fluorine substituents can stabilizes the HOMO effectively, while the LUMO is affected much less. Moreover, the ancillary ligand L-alanine has little contribution to HOMOs and LUMOs. These facts are consistent with the conclusions obtained in the electrochemical experiments. According to TD-DFT calculation, the low-energy absorption bands (320–550 nm) in the electron absorption spectra are mainly generated by HOMO → LUMO and HOMO → LUMO + 1 transitions, which are assigned to a mixture of MLCT and LLCT (Figure 4, Table 1, and Tables S1–S6).

### 2.5. Cation-Binding Properties

**Figure 5:**
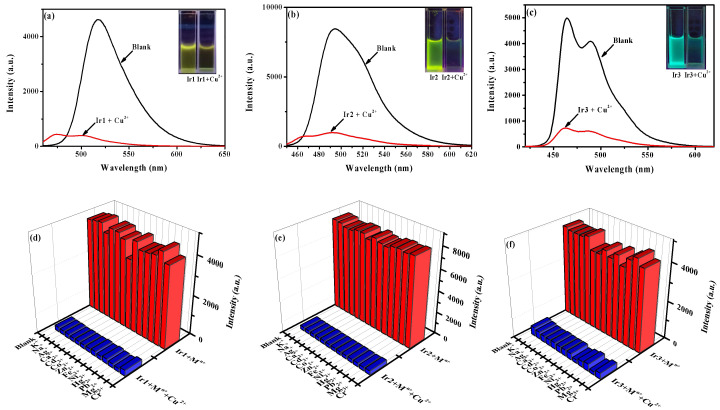
Phosphorescent emission spectra and luminescent color (illustration) changes in acetonitrile solution of complexes (**a**) **Ir1**, (**b**) **Ir2**, and (**c**) **Ir3** (20 μmol/L) before and after adding 1.0 equiv. Cu^2+^. Competitive tests on the phosphorescence responses of complexes (**d**) **Ir1**, (**e**) **Ir2,** and (**f**) **Ir3** to various metal cations (*I*_509_ for **Ir1**, *I*_493_ for **Ir2**, *I*_464_ for **Ir3**). Red columns represent the addition of various metal ions (10.0 equiv.) to the blank solution and blue columns represent the addition of Cu^2+^ (10.0 equiv.) to the solutions with various metal ions.

The photoluminescence response specificity of complexes **Ir1**–**Ir3** (50 mmol/L) toward Cu^2+^ over other metal ions, including K^+^, Na^+^, Ag^+^, Ca^2+^, Cd^2+^, Co^2+^, Zn^2+^, Fe^2+^, Ni^2+^, Hg^2+^, Pb^2+^, Mg^2+^, and Cr^3+^, were systematically investigated using photoluminescence spectroscopy in acetonitrile solution. When the above metal ions (10.0 equiv.) were added to the solution of complexes **Ir1**–**Ir3**, the luminescence was quenched obviously only after the addition of Cu^2+^, whereas the addition of other cations caused tiny luminescence changes (Figures S7–S9). The spectral emission intensity of complexes **Ir1**–**Ir3** were decreased by 94–96% by adding Cu^2+^. Moreover, the existence of Cu^2+^ ions were converted into a visual signal by **Ir1**–**Ir3** that could be seen with the naked-eye under 365 UV light (Figure 5a–c).

The metal ions competitive experiments were carried out to study the anti-interference of complexes **Ir1**–**Ir3** in sensing Cu^2+^, and the results are recorded in Figure 5d–f. For complexes **Ir1**–**Ir3**, the emission intensities were greatly quenched when 10.0 equiv. Cu^2+^ ions were added into the mixing solutions of Ir(III) complexes and coexisting metal ions, which are consistent with those obtained by adding Cu^2+^ ions alone to the solutions of iridium complexes. The results indicated that complexes **Ir1**–**Ir3** can specifically discriminate Cu^2+^ from other metal ions and show an obvious “turn-off” response, indicating its good selectivity and anti-interference ability.

As shown in Figure S12, when Na_2_EDTA was added to the mixture solution of Ir(III) complex and Cu^2+^ ions, the emission intensities of complexes **Ir1**–**Ir3** were nearly entirely restored in two minutes. The results indicate that the free Ir(III) complexes can be regenerated by the addition of Na_2_EDTA to the mixture of Ir(III) complexes and Cu^2+^ ions due to the high affinity between Na_2_EDTA and the complexes, suggesting that the detection mechanism of Ir(III) complexes for Cu^2+^ may be binding-based mechanisms. Then, ^1^H NMR spectrum of **Ir2** with 1.0 equiv. Cu^2+^ in DMSO-*d*_6_ was measured (Figure S13). It was found by analysis that the proton signal at 8.57 ppm corresponded to the H_b_ of pyridyl ring disappeared, and the other proton signals did not change significantly. We speculate that the detection mechanism of the complex may be due to the coordination between Cu^2+^ and the oxygen atom of L-alanine, which leads to the luminescence quenching of the Ir(III) complex by the paramagnetic effect from the spin-orbit coupling of the Cu^2+^. [27] The possible working mechanism of Ir(III) complex on Cu^2+^ detection is shown in Scheme S1.

**Figure 6:**
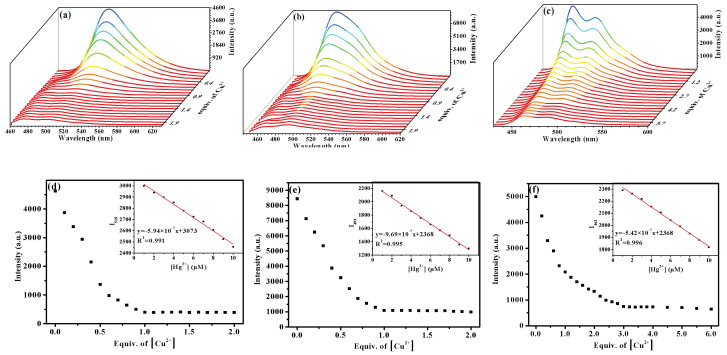
Changes in phosphorescent emission spectra of (**a**) **Ir1**, (**b**) **Ir2**, and (**c**) **Ir3** (20 μmol/L) in acetonitrile with various amounts of Cu^2+^ (0–20 μmol/L). Plot of the luminescence intensity of complexes (**d**) **Ir1,** (**e**) **Ir2,** and (**f**) **Ir3** against the concentration of Cu^2+^ in acetonitrile (20 μmol/L).

In order to further evaluate the linear relationship between the photoluminescence response of complexes **Ir1**–**Ir3** and the amount of Cu^2+^ added, emission titration experiments were investigated (Figure 6a–c). It was found that the photoluminescence intensity of complexes **Ir1**–**Ir3** at their strongest emission peaks decreased along with the additional amount of Cu^2+^, and then gradually reached a plateau (Figure 6d–f). In a certain range, the luminescence intensity of complexes **Ir1**–**Ir3** had a good linear relationship with copper ion concentration. According to the titration curves of complexes **Ir1**–**Ir3** with Cu^2+^, the complexation constant (K) of **Ir1**–**Ir3** with Cu^2+^ was determined by using the Benesi–Hildebrand equation [28], and the K value was 5.0 × 10^4^ L/mol, 4.9 × 10^4^ L/mol, and 2.9 × 10^4^ L/mol. According to the detection limit calculation formula, 3*σ*/*k*, where *σ* is the standard deviation of blank measurement, *k* is the slope of emission intensity of complex and Cu^2+^ titration curve, the detection limit of **Ir1**–**Ir3** calculated is 1.9 × 10^−6^ mol/L, 1.1 × 10^−6^ mol/L, and 2.0 × 10^−6^ mol/L, which are lower than the maximum allowable copper content in drinking water 30 μmol/L set by the World Health Organization (WHO) [29]. 

### 2.6. Water Sample Analysis

In order to investigate the application ability of complexes **Ir1**–**Ir3** towards Cu^2+^ in real samples, three water samples including tap, drinking, and lake water were analyzed by the spike-and-recovery method [30]. To remove large particles, all water samples were filtered through a 0.2 mm membrane. Cu^2+^ was added into the water sample to make the Cu^2+^ concentration 2.0 μmol/L, 4.0 μmol/L, and 6.0 μmol/L. The experimental results are shown in Table 3, Table 4 and Table 5. The recovery rates of Cu^2+^ for complexes **Ir1**–**Ir3** in water samples are 83–121%, 90–111%, and 95–101%, respectively. These results indicate that complexes **Ir2** and **Ir3** containing fluorinated substituents show better recovery rates, and they have potential applications in monitoring the concentrations of Cu^2+^ in real samples.

## 3. Materials and Methods

### 3.1. Instruments

The chemicals used in this paper are all analytical grade reagents. ^1^H and ^13^C NMR spectra were obtained by Bruker AM 400 MHz spectrometers using deuterated dimethyl sulfoxide (DMSO-*d*_6_) and deuterated chloroform (CDCl_3_) as recording solvents. ^19^F NMR spectra were obtained by Bruker AVANCE NEO 600 MHz using DMSO-*d*_6_ as recording solvents. Mass spectra (MS) were obtained with ESI-MS (Agilent 6520Q-TOF LC/MS). Absorption spectra and photoluminescence spectra were measured by a UV-2700 spectrophotometer and Hitachi F-2700 spectrophotometer, respectively. The decay lifetimes of the complexes in deoxygenated acetonitrile solution were determined by an FLS920P fluorescence spectrometer. Cyclic voltammetry was performed on a CHI 760E electrochemical workstation with platinum thread; AgNO_3_ (0.010 mol/L in CH_3_CN)-Ag and a polished Pt plate were used as the counter electrode, reference electrode, and working electrode, respectively. Both density functional theory (DFT) and time-dependent DFT (TD-DFT) were carried out using the Gaussian 09 software package [31,32]. The PLQYs of the complex were calculated according to Equation (1) [33].

(1) $$\Phi_{\mathrm{unk}}=\Phi_{\mathrm{std}}(\frac{I_{\mathrm{unk}}}{I_{\mathrm{std}}})(\frac{A_{\mathrm{std}}}{A_{\mathrm{unk}}})(\frac{\eta_{\mathrm{unk}}}{\eta_{\mathrm{std}}}{)}^{2}$$

where *Φ*_unk_, *I*_unk_, and *A*_unk_ represent the luminescent quantum yield, integrated emission intensities, and absorbance under excitation wavelength of unknown samples, respectively. *Φ*_std_, *I*_std_, and *A*_std_ stand for Ir(ppy)_3_. *η*_unk_ and *η*_std_ represent the pure solvent refractive indices. The *Φ*_std_ of Ir(ppy)_3_ in room temperature is known to be 0.97 (error: ±10%) [34]. 

### 3.2. Synthesis

Cyclometalated ligands 2-phenylpyridine (ppy), 2-(4-fluorophenyl)pyridine (F_4_ppy), 2-(2,4,5-trifluorophenyl)pyridine (F_2,4,5_ppy) [35], and Ir(III) chloro-bridged dimers were synthesized according to the literature [36]. 

L-alanine (2.5 equiv.), potassium tert-butoxide (2.5 equiv.), and iridium(III) chloride bridged dimers (1.0 equiv.) were refluxed for 12 h at 140 °C in DMF (30 mL) under nitrogen atmosphere. The solvent was evaporated under pressure and the coarse product was obtained. Then, silica gel column chromatography was used to purify the crude product to obtain the target complexes **Ir1**–**Ir3.**

**(ppy)_2_Ir(L-alanine) (Ir1)**: 0.15 g (1.7 mmol) L-alanine and 0.47 g (0.68 mmol) [(ppy)_2_Ir(μ-Cl)]_2_ obtained 0.57 g of **Ir1** as a yellow solid with 51% yield. ^1^H NMR (400 MHz, DMSO-*d*_6_): δ 9.12 (dd, *J* = 38.6, 5.8 Hz, 1H), 8.61 (dd, *J* = 23.4, 5.8 Hz, 1H), 8.16 (q, *J* = 8.2 Hz, 2H), 7.94 (q, *J* = 7.0 Hz, 2H), 7.76–7.67 (m, 2H), 7.42 (dt, *J* = 26.4, 6.3 Hz, 2H), 6.76 (q, *J* = 8.0 Hz, 2H), 6.60 (t, *J* = 7.3 Hz, 2H), 6.31–6.20 (m, 1H), 5.95 (t, *J* = 7.1 Hz, 1H), 3.82 (t, *J* = 10.9 Hz, 1H), 1.26 (t, *J* = 7.1 Hz, 1H), 1.18 (d, *J* = 7.0 Hz, 2H). ^13^C NMR (101 MHz, DMSO-*d*_6_) δ 174.12, 173.14, 168.31, 167.68, 150.54, 149.88, 148.57, 148.32, 144.96, 144.48, 138.56, 132.50, 131.97, 129.82, 129.41, 125.09, 124.60, 123.64, 123.38, 121.57, 120.98, 119.75, 119.61, 66.80, 65.89. MS (ESI) m/z: calcd for [C_25_H_22_IrN_3_O_2_Na]^+^, 612.1316; found, 612.1341 [M + Na]^+^.

**(F_4_ppy)_2_Ir(L-alanine) (Ir2):** 0.15 g (1.7 mmol) L-alanine and 0.50 g (0.68 mmol) [(F_4_ppy)_2_Ir(μ-Cl)]_2_ obtained 0.44 g of **Ir2** as a yellow solid with 52% yield. ^1^H NMR (400 MHz, DMSO-*d*_6_) δ 9.08 (dd, *J* = 39.9, 5.4 Hz, 1H), 8.57 (dd, *J* = 19.9, 5.5 Hz, 1H), 8.18 (t, *J* = 8.3 Hz, 2H), 8.03–7.94 (m, 2H), 7.88–7.78 (m, 2H), 7.46 (dd, *J* = 24.0, 6.1 Hz, 2H), 6.62 (d, *J* = 6.9 Hz, 2H), 5.83 (d, *J* = 10.0 Hz, 1H), 5.51 (t, *J* = 8.1 Hz, 1H), 3.08 (d, *J* = 8.0 Hz, 1H), 1.22 (dd, *J* = 36.7, 7.0 Hz, 3H). ^13^C NMR (101 MHz, DMSO-*d*_6_): δ 182.92, 182.20, 167.43, 167.33, 167.00, 166.72, 163.57, 163.35, 161.08, 160.86, 157.26, 157.22, 156.86, 151.10, 151.04, 150.51, 150.14, 147.55, 147.22, 141.43, 141.28, 137.89, 50.46, 48.57, 21.23. ^19^F NMR (565 MHz, DMSO-*d*_6_) δ −110.81, −111.48. MS (ESI) m/z: calcd for [C_25_H_20_F_2_IrN_3_O_2_Na]^+^, 647.6602; found, 647.6625 [M + Na]^+^.

**(F_2,4,5_ppy)_2_Ir(L-alanine) (Ir3):** 0.15 g (1.7 mmol) L-alanine and 0.52 g (0.68 mmol) [(F_2,4,5_ppy)_2_Ir(μ-Cl)]_2_ obtained 0.54 g of **Ir3** as a yellow solid with 55% yield. ^1^H NMR (400 MHz, DMSO-*d*_6_): δ 9.14 (d, *J* = 26.3 Hz, 1H), 8.63 (s, 1H), 8.24 (s, 2H), 8.05 (s, 2H), 7.44 (s, 2H), 7.00 (s, 2H), 3.06 (s, 1H), 1.20 (d, *J* = 46.9 Hz, 3H). ^13^C NMR (101 MHz, DMSO-*d*_6_): δ 183.72, 183.15, 165.69, 165.42, 164.55, 164.53, 157.81, 154.90, 152.46, 152.02, 149.31, 148.33, 139.14, 137.58, 137.04, 132.63, 131.98, 131.08, 130.62, 123.10, 100.44, 99.52, 55.46, 48.41, 40.89, 21.54. ^19^F NMR (565 MHz, DMSO-*d*_6_) δ −115.74 (*J* = 376.5, 18.3 Hz), −133.33 (d, *J* = 26.8 Hz), −133.74 (t, *J* = 29.0 Hz), −134.53 (dd, *J* = 39.2, 18.6 Hz), −137.55 (d, *J* = 6.1 Hz), −137.77 (d, *J* = 6.8 Hz). MS (ESI) m/z: calcd for [C_25_H_16_F_6_IrN_3_O_2_Na]^+^, 719.6225; found, 719.6234 [M + Na]^+^.

### 3.3. Cation-Binding Properties

A 0.1 mol/L aqueous solution of Cu^2+^ and other metal ions (K^+^, Na^+^, Ag^+^, Ca^2+^, Cd^2+^, Co^2+^, Zn^2+^, Fe^2+^, Ni^2+^, Hg^2+^, Pb^2+^, Mg^2+^ and Cr^3+^) was prepared using the corresponding metal nitrate. Various metal ions were added into the solution of **Ir1**–**Ir3** (20 μmol/L) for selective experiments by monitoring the changes of photoluminescence intensity. The cation competition was studied by adding 10.0 equiv. Cu^2+^ into the mixed solution of Ir(III) complexes and other metal ions. In titration experiments, different concentrations of Cu^2+^ ions were gradually added to the solution of **Ir1**–**Ir3** and each time the spectral changes were measured.

## 4. Conclusions

A series of new luminescent cyclometalated Ir(III) complexes with L-alanine as the ancillary ligand were synthesized, and the electronic absorption, photophysical, and electrochemical properties of these complexes were investigated. All complexes showed strong phosphorescence in acetonitrile solution at room temperature. The luminescent color of the complexes **Ir1**–**Ir3** changed from yellow to sky blue and the PLQYs increased from 0.48 to 0.69 with the increase in fluorine substituents on the cyclometalated ligands. DFT calculations show that the energy of HOMOs and LUMOs can be adjusted and controlled by increasing the number of fluorine substituents on the cyclometalated ligands, which provides a basis for designing efficient iridium complexes with different luminescence colors. Complexes **Ir1**–**Ir3** show a rapid photoluminescence quenching response after adding Cu^2+^, and exhibit good selectivity in acetonitrile solution with detection limits of 1.9 × 10^−6^ mol/L, 1.1 × 10^−6^ mol/L, and 2.0 × 10^−6^ mol/L, respectively. In the test of water samples, the complexes **Ir1**–**Ir3** showed a good recovery rate with 83–121%, 90–111%, and 95–101%. Therefore, the iridium complexes reported in this work can be used as efficient chemosensors for the detection of Cu^2+^.

## Figures and Tables

**Table 1:** Photophysical and electrochemical data for complexes **Ir1**–**Ir3**.

Complex	*λ*_abs_ (nm) ^a^	*λ*_em_ (nm) ^b^	PLQY	*k*_r_ (10^5^ s^−1^)	*k*_nr_ (10^5^ s^−1^)	*τ* (μs)	*E*_OX_ (V) ^c^	*E*_HOMO_ (eV) ^d^	*E*_red_ (V) ^c^	*E*_LUMO_ (eV) ^d^
**Ir1**	262, 404, 454	509	0.48	2.9	3.2	1.64	0.59	−5.19	−1.07	−3.70
**Ir2**	258, 380, 423	493	0.55	3.3	2.7	1.67	0.73	−5.33	−1.08	−3.52
**Ir3**	250, 311, 378	464, 490	0.69	4.7	2.1	1.46	0.91	−5.51	−1.07	−3.53

^a, b^ Measured in acetonitrile at room temperature with the concentration of phosphors at 2.0 × 10^−5^ mol/L. ^c^ Measured in degassed DCM: MeCN [1:1(*v*/*v*)] at a scan rate of 0.10 V·s^−1^ versus Fc^+^/Fc using 0.10 mol/L [*n*-Bu_4_N]PF_6_ as a supporting electrolyte. ^d^ *E*_HOMO_ = −[*E*_ox_ − *E*_(Fc_^+^_/Fc)_ + 4.8] eV; *E*_LUMO_ = −[*E*_red_ − *E*_(Fc_^+^_/Fc)_ + 4.8] eV.

**Table 2:** The frontier orbital energy and electron density distribution for **Ir1**–**Ir3**.

Complex	Orbital	Energy (eV) (Calculated)	*E*_g_ (eV) (Calculated)	Composition %			
				Ir	Cyclometalated ligands		Ancillary ligands
					phenyl group	pyridyl group	
**Ir1**	HOMO	−5.22	3.65	52.28	35.88	6.24	2.60
	LUMO	−1.52		4.79	26.68	66.42	2.12
**Ir2**	HOMO	−5.37	3.80	50.50	36.23	7.57	5.71
	LUMO	−1.58		4.76	25.91	67.20	2.13
**Ir3**	HOMO	−5.64	3.96	50.82	38.30	4.62	6.26
	LUMO	−1.69		4.49	26.85	66.01	2.65
	LUMO+1	−1.68		5.05	27.71	65.26	1.99

**Table 3:** Application in real samples testing for **Ir1**.

Sample	[Cu^2+^] (μmol/L)	Found [Cu^2+^] (μmol/L)	Recovery (%)
Lake water	2.0	2.4	83.3
Lake water	4.0	3.3	121
Lake water	6.0	5.6	107
Tap water	2.0	2.4	83.3
Tap water	4.0	3.6	111
Tap water	6.0	5.5	109
Drinking water	2.0	2.3	87.0
Drinking water	4.0	3.5	114
Drinking water	6.0	5.7	105

**Table 4:** Application in real samples testing for **Ir2**.

Sample	[Cu^2+^] (μmol/L)	Found [Cu^2+^] (μmol/L)	Recovery (%)
Lake water	2.0	2.0	100
Lake water	4.0	3.9	102
Lake water	6.0	5.7	105
Tap water	2.0	2.0	100
Tap water	4.0	4.0	100
Tap water	6.0	5.5	109
Drinking water	2.0	1.8	111
Drinking water	4.0	4.4	90.9
Drinking water	6.0	6.1	98.3

**Table 5:** Application in real samples testing for **Ir3**.

Sample	[Cu^2+^] (μmol/L)	Found [Cu^2+^] (μmol/L)	Recovery (%)
Lake water	2.0	1.9	105
Lake water	4.0	4.0	100
Lake water	6.0	6.1	98.3
Tap water	2.0	2.1	95.2
Tap water	4.0	4.2	95.2
Tap water	6.0	5.9	101
Drinking water	2.0	2.1	95.2
Drinking water	4.0	4.1	97.5
Drinking water	6.0	6.1	98.3

## Data Availability

The data presented in this study are available in supplementary material.

## Supplementary Materials

The following supporting information can be downloaded at: https://www.mdpi.com/article/10.3390/molecules27238506/s1, Figure S1: The ^1^H NMR spectrum of the complex **Ir1**; Figure S2: The ^13^C NMR spectrum of the complex **Ir1.**; Figure S3: The ^1^H NMR spectrum of the complex **Ir2**; Figure S4: The ^13^C NMR spectrum of the complex **Ir2**; Figure S5: The ^1^H NMR spectrum of the complex **Ir3**; Figure S6: The ^13^C NMR spectrum of the complex **Ir3**; Figure S7: The ^19^F NMR spectrum of the complex **Ir2**; Figure S8: The ^19^F NMR spectrum of the complex **Ir3**; Figure S9: Photoluminescence spectra of **Ir1** upon addition of various metal ions (10 equiv.) in acetonitrile solutions; Figure S10: Photoluminescence spectra of **Ir2** upon addition of various metal ions (10 equiv.) in acetonitrile solutions; Figure S11: Photoluminescence spectra of **Ir3** upon addition of various metal ions (10 equiv.) in acetonitrile solutions; Figure S12: The emission spectra of **Ir1**–**Ir3** in the absence of Cu^2+^ (black line), in the presence of Cu^2+^ (red line) and in presence of both Cu^2+^ and Na_2_EDTA (blue line) in CH_3_CN. (a) **Ir1** (b) **Ir2** (c) **Ir3**; Figure S13: ^1^H NMR (DMSO-*d*_6_) spectra of complex **Ir2** (red line) and **Ir2** + 1.0 equiv. of Cu^2+^ (green line); Scheme S1: Possible sensing mechanism of **Ir2** with Cu^2^+; Table S1: Distribution of energy and electron density along the orbit of frontier molecules of **Ir1**; Table S2: Distribution of energy and electron density along the orbit of frontier molecules of **Ir2**; Table S3: Distribution of energy and electron density along the orbit of frontier molecules of **Ir3**; Table S4: Summary of the results of TD-DFT calculations on complex **Ir1** (assignment is provided for MO contributions > 10%); Table S5: Summary of the results of TD–DFT calculations on complex **Ir2** (assignment is provided for MO contributions > 10%); Table S6: Summary of the results of TD–DFT calculations on complex **Ir3** (assignment is provided for MO contributions > 10%).

## Sample Availability

Samples of the compounds **Ir1**–**Ir3** are available from the authors.
