# Supplementary material for: Multicolor Emissive Phosphorescent Iridium(III) Complexes Containing L-Alanine Ligands: Photophysical and Electrochemical Properties, DFT Calculations, and Selective Recognition of Cu(II) Ions
Source: Molecules. 2022 Dec 3;27(23):8506. doi: 10.3390/molecules27238506 (PMC9741083; doi:10.3390/molecules27238506)
Supplement: Supplementary file 1 [file molecules-27-08506-s001.zip › molecules-2060697-supplementary.pdf]

Supporting Information

Multicolor emissive phosphorescent iridium(III) complexes containing L-alanine ligands: Photophysical and electrochemical properties, DFT calculations, and selective recognition of Cu(II) ions

Xi Chu, Yichuan Huang, Wenhao Li, Shisheng Zhao, Hongyan Li*, Aidang Lu*

School of Chemical Engineering and Technology, Hebei University of Technology, Tianjin 300130,

China

* Correspondence: hyli@hebut.edu.cn (H.L.), luaidang@hebut.edu.cn (A.L.)

Table of Contents

Figure S1. The ^{1}H NMR spectrum of the complex **Ir1**.

Figure S2. The ^{13}C NMR spectrum of the complex **Ir1.**

Figure S3. The ^{1}H NMR spectrum of the complex **Ir2**.

Figure S4. The ^{13}C NMR spectrum of the complex **Ir2**.

Figure S5. The ^{1}H NMR spectrum of the complex **Ir3**.

Figure S6. The ^{13}C NMR spectrum of the complex **Ir3**.

Figure S7. The ^{19}F NMR spectrum of the complex **Ir2**.

Figure S8. The ^{19}F NMR spectrum of the complex **Ir3**.

Figure S9. Photoluminescence spectra of **Ir1** upon addition of various metal ions (10 equiv.) in acetonitrile solutions.

Figure S10. Photoluminescence spectra of **Ir2** upon addition of various metal ions (10 equiv.) in acetonitrile solutions.

Figure S11. Photoluminescence spectra of **Ir3** upon addition of various metal ions (10 equiv.) in acetonitrile solutions.

Figure S12. The emission spectra of **Ir1–Ir3** in the absence of Cu^{2+} (black line), in the presence of Cu^{2+} (red line) and in presence of both Cu^{2+} and Na_2EDTA (blue line) in CH_3CN. (a) **Ir1** (b) **Ir2** (c) **Ir3**

Figure S13. ^{1}H NMR (DMSO-d_6) spectra of complex **Ir2** (red line) and **Ir2** + 1.0 equiv. of Cu^{2+} (green line).

Scheme S1. Possible sensing mechanism of **Ir2** with Cu^{2+}.

Table S1. Distribution of energy and electron density along the orbit of frontier molecules of **Ir1**.

Table S2. Distribution of energy and electron density along the orbit of frontier molecules of **Ir2**.

Table S3. Distribution of energy and electron density along the orbit of frontier molecules of **Ir3**.

Table S4. Summary of the results of TD−DFT calculations on complex **Ir1** (assignment is provided for MO contributions＞10 %)

Table S5. Summary of the results of TD−DFT calculations on complex **Ir2**

(assignment is provided for MO contributions＞10 %)

Table S6. Summary of the results of TD−DFT calculations on complex **Ir3** (assignment is provided for MO contributions＞10 %)

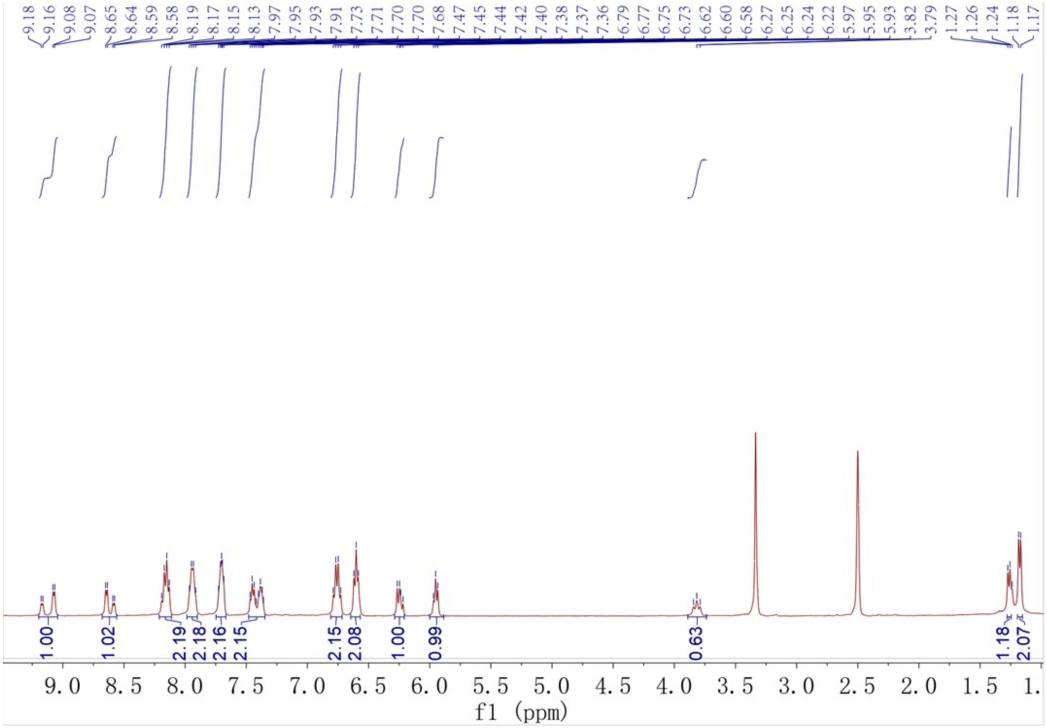

Figure S1. The ^{1}H NMR spectrum of the complex **Ir1**.

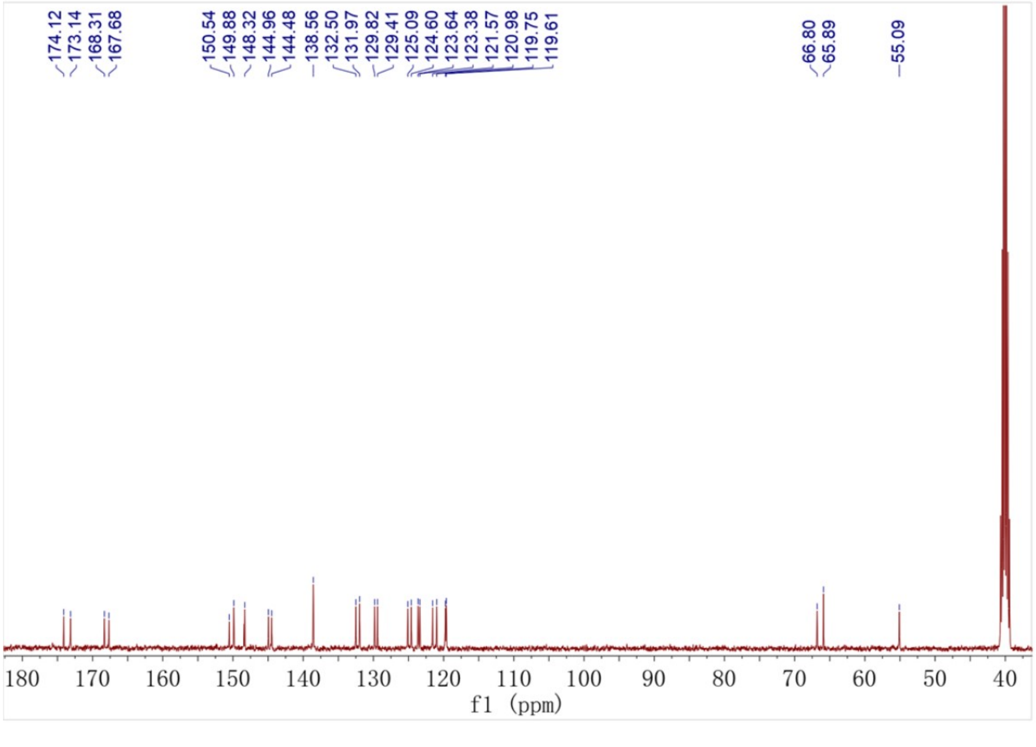

Figure S2. The ^{13}C NMR spectrum of the complex **Ir1.**

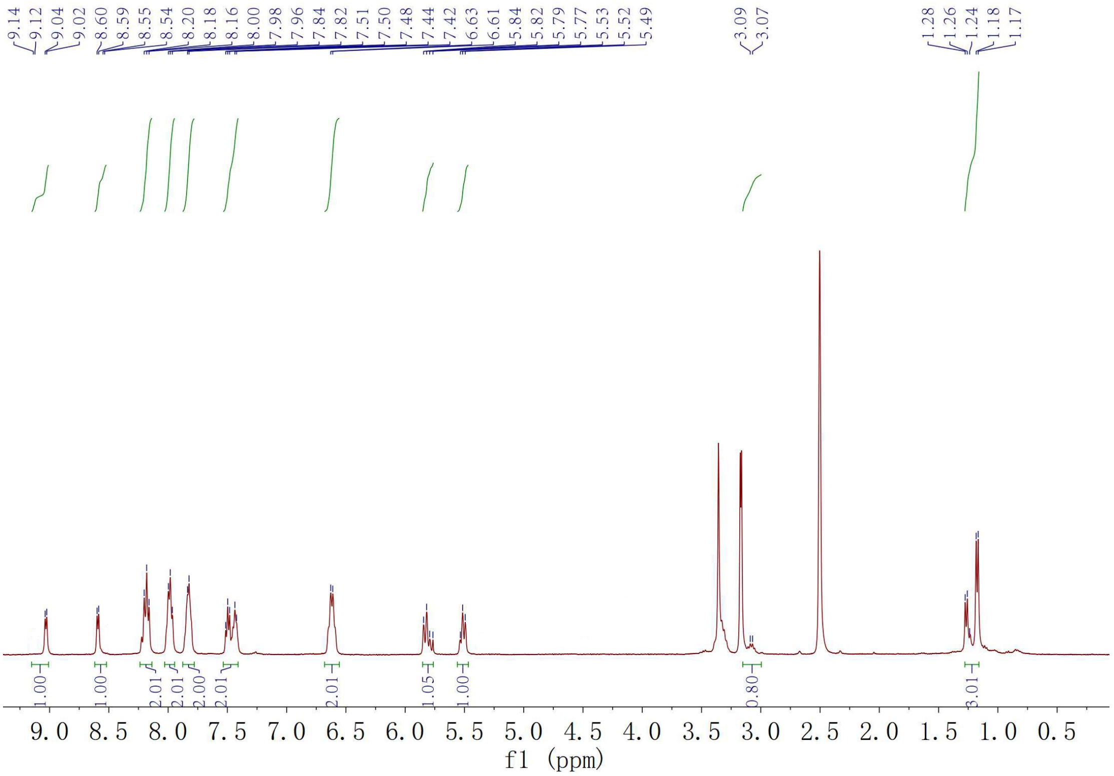

Figure S3. The ^{1}H NMR spectrum of the complex **Ir2**.

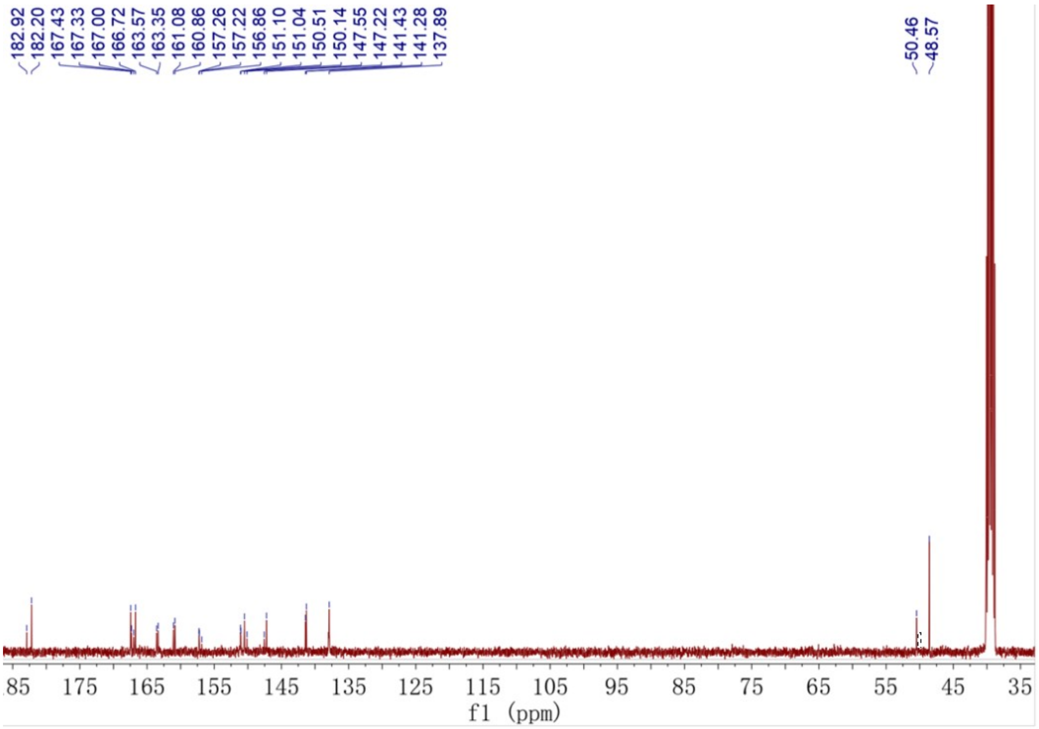

Figure S4. The ^{13}C NMR spectra of the complex **Ir2**.

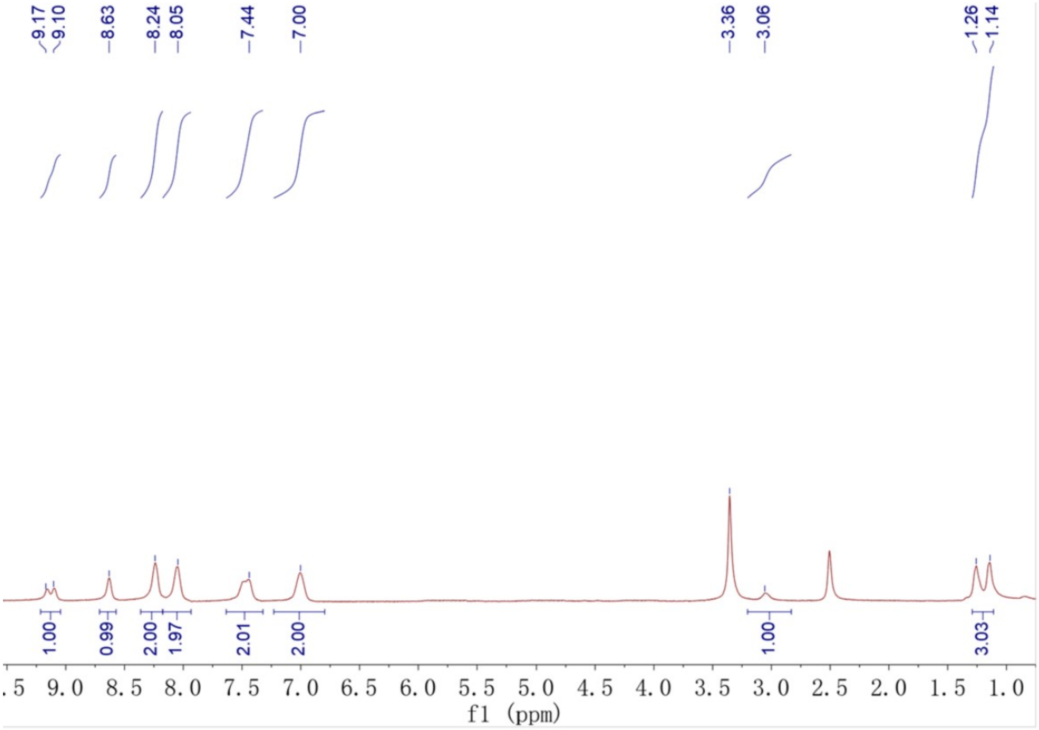

Figure S5. The ^{1}H NMR spectrum of the complex **Ir3**.

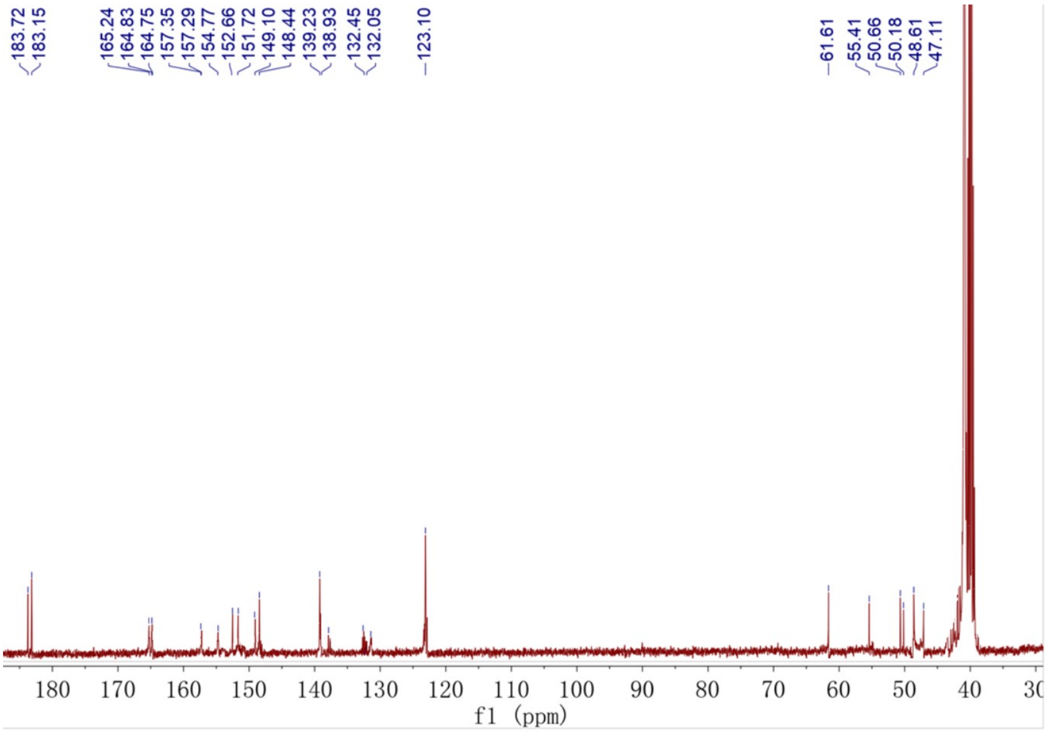

Figure S6. The ^{13}C NMR spectrum of the complex **Ir3**.

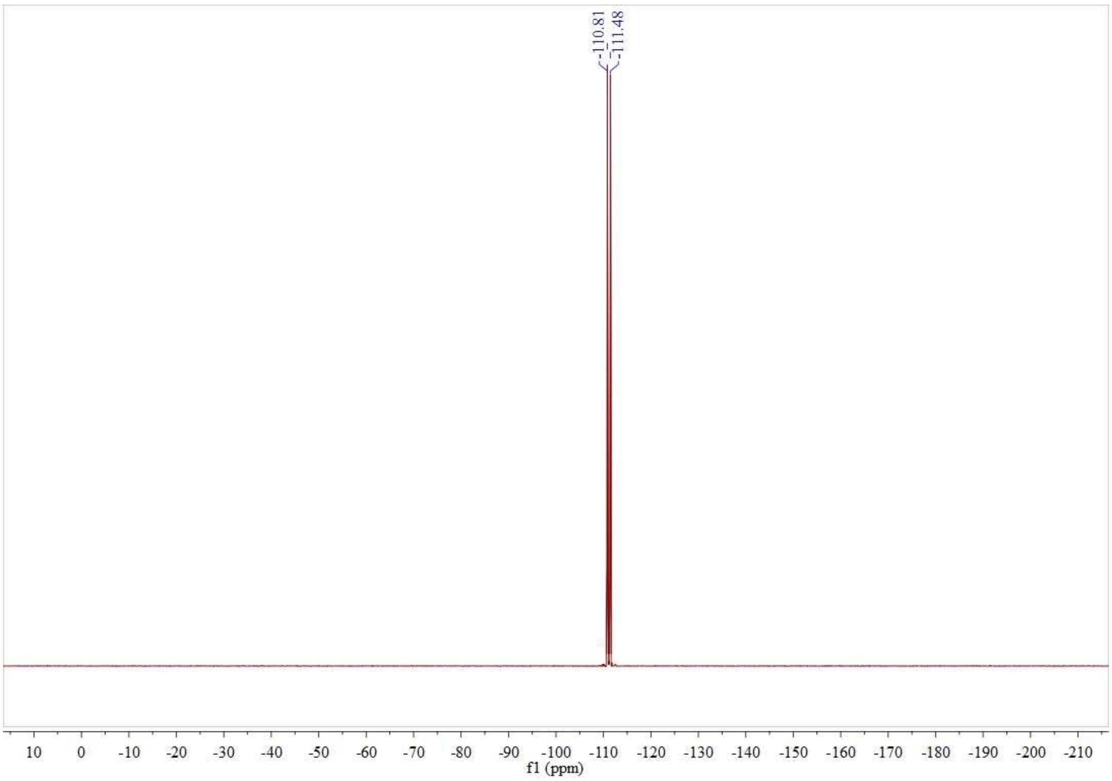

Figure S7. The ^{19}F NMR spectrum of the complex **Ir2**.

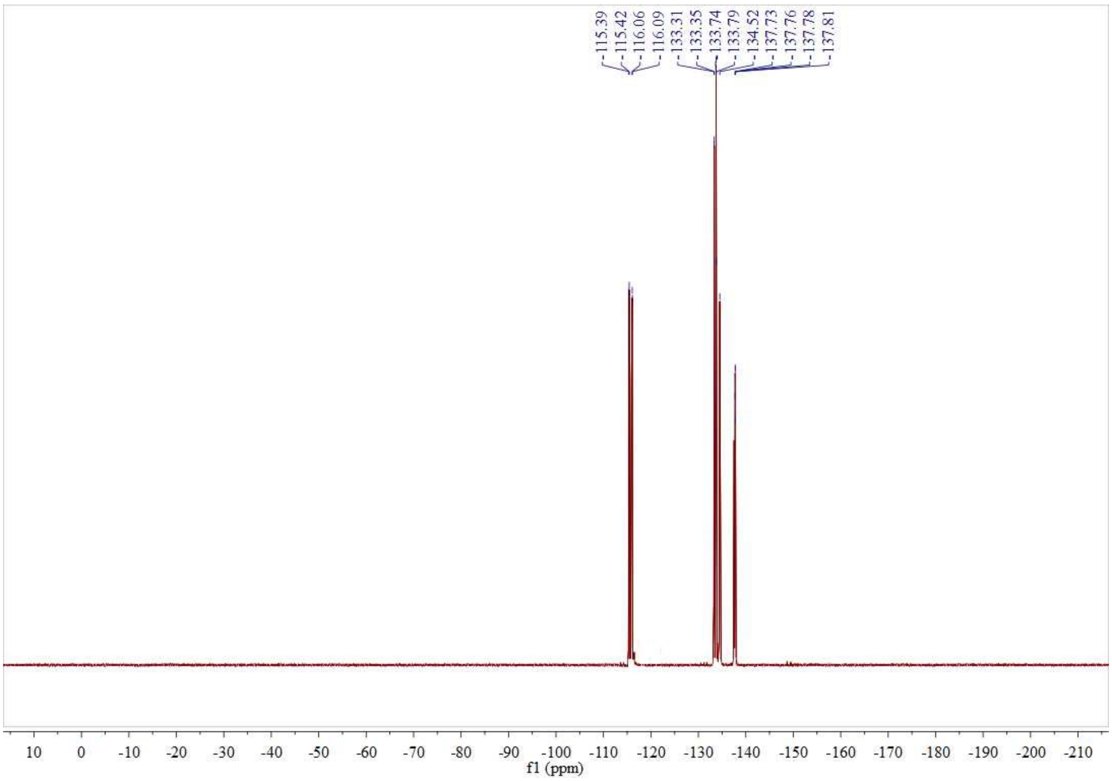

Figure S8. The ^{19}F NMR spectrum of the complex **Ir3**.

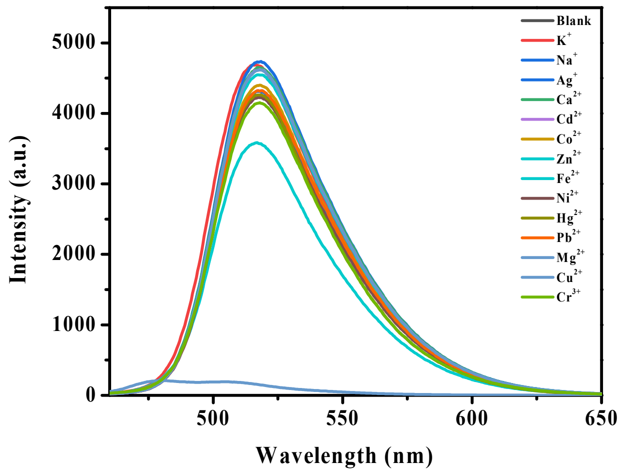

Figure S9. Photoluminescence spectra of **Ir1** upon addition of various metal ions (10.0 equiv.) in acetonitrile solutions.

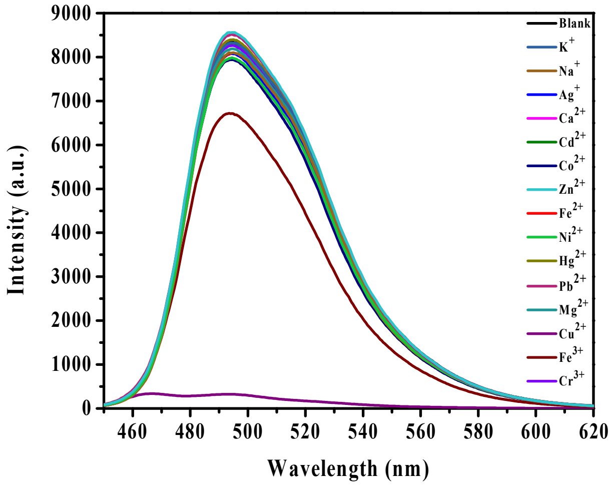

Figure S10. Photoluminescence spectra of **Ir2** upon addition of various metal ions (10.0 equiv.) in acetonitrile solutions.

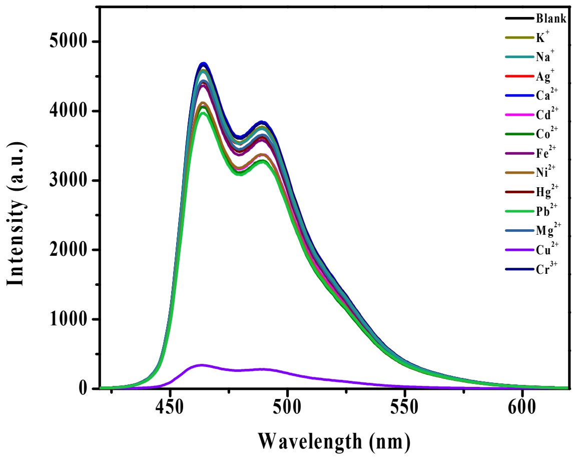

Figure S11. Photoluminescence spectra of **Ir3** upon addition of various metal ions (10.0 equiv.) in acetonitrile solutions.

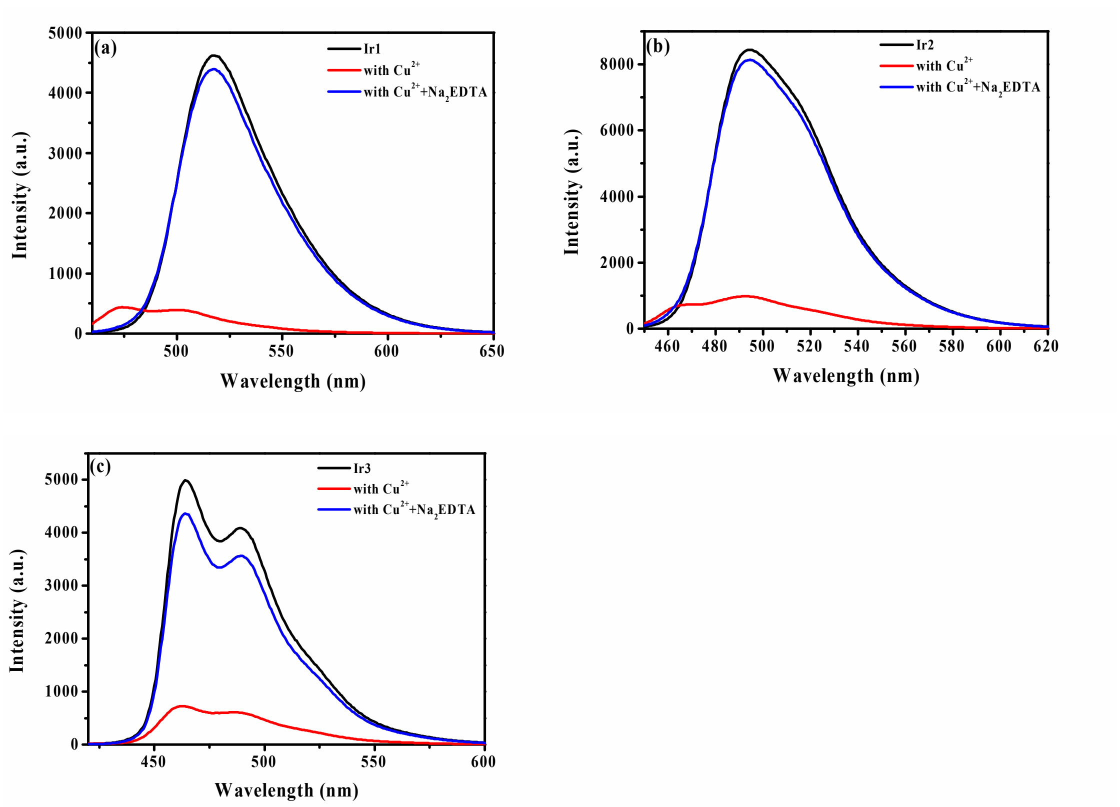

Figure S12. The emission spectra of **Ir1–Ir3** in the absence of Cu^{2+} (black line), in the presence of Cu^{2+} (red line) and in the presence of both Cu^{2+} and Na_2EDTA (blue line) in CH_3CN solution. (a) **Ir1** (b) **Ir2** (c) **Ir3.**

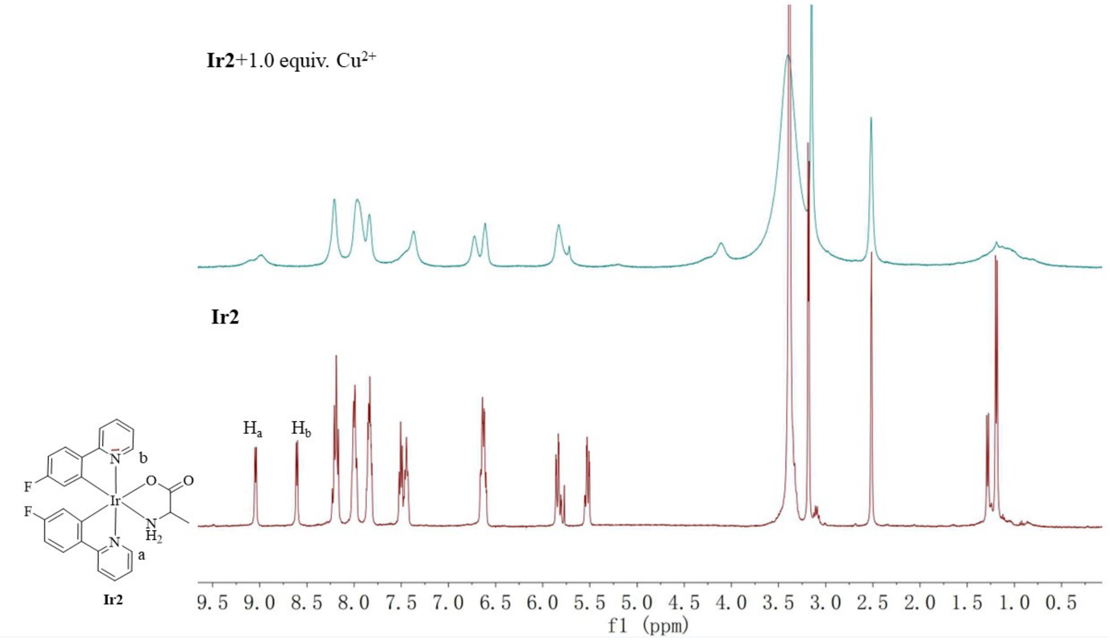

Figure S13. ^{1}H NMR (DMSO-d_6) spectra of complex **Ir2** (red line) and **Ir2** + 1.0 equiv. of Cu^{2+} (green line).

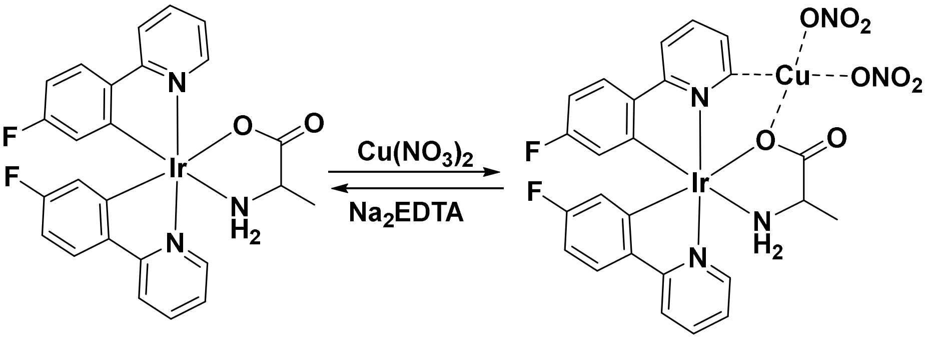

Scheme S1. Possible sensing mechanism of **Ir2** with Cu^{2+}.

Table S1. Distribution of energy and electron density along the orbit of frontier molecules of **Ir1**.

**Complex**	**Orbital**	**Energy (eV) (Calculated)**	**Composition %**			
			Ir	Cyclometalated ligands		Ancillary ligands
				phenyl	pyridyl	
**Ir1**	HOMO−5	−6.439	6.10	20.88	6.65	66.38
	HOMO−4	−6.415	16.07	56.30	24.25	3.38
	HOMO−3	−6.213	57.19	23.92	12.70	6.18
	HOMO−2	−6.135	18.55	50.19	23.41	7.85
	HOMO−1	−5.869	63.52	9.20	11.13	16.15
	HOMO	−5.217	52.28	35.88	6.24	5.60
	LUMO	−1.571	4.79	26.68	66.42	2.12
	LUMO+1	−1.519	5.05	22.43	70.27	2.25
	LUMO+2	−1.047	3.23	12.91	80.99	2.86
	LUMO+3	−0.943	3.31	10.94	83.72	2.03
	LUMO+4	−0.153	10.92	72.99	5.33	10.77

Table S2. Distribution of energy and electron density along the orbit of frontier molecules of **Ir2**.

**Complex**	**Orbital**	**Energy (eV) (Calculated)**	**Composition %**			
			Ir	Cyclometalated ligands		Ancillary ligands
				phenyl	pyridyl	
**Ir2**	HOMO−5	−6.491	4.01	16.11	2.52	77.36
	HOMO−4	−6.427	49.07	31.57	16.05	3.30
	HOMO−3	−6.309	41.09	34.93	16.46	7.52
	HOMO−2	−6.144	16.42	52.27	23.72	7.59
	HOMO−1	−5.954	57.88	13.80	12.77	15.55
	HOMO	−5.372	50.50	36.23	7.57	5.71
	LUMO	−1.581	4.76	25.91	67.20	2.13
	LUMO+1	−1.534	4.83	22.07	70.91	2.19
	LUMO+2	−1.084	3.16	15.56	78.45	2.83
	LUMO+3	−0.973	3.33	12.04	82.63	2.00
	LUMO+4	−0.061	8.08	76.69	6.86	8.36

Table S3. Distribution of energy and electron density along the orbit of frontier molecules of **Ir3.**

**Complex**	**Orbital**	**Energy (eV) (Calculated)**	**Composition %**			
			Ir	Cyclometalated ligands		Ancillary ligands
				phenyl	pyridyl	
**Ir3**	HOMO−5	−6.655	34.72	8.19	9.56	48.74
	HOMO−4	−6.547	21.67	37.90	10.22	31.05
	HOMO−3	−6.392	17.62	50.60	21.91	10.01
	HOMO−2	−6.294	10.59	67.16	21.46	0.82
	HOMO−1	−6.144	48.87	24.21	11.95	15.20
	HOMO	−5.641	50.82	38.30	4.62	6.57
	LUMO	−1.686	4.49	26.85	66.01	2.68
	LUMO+1	−1.682	5.05	27.71	65.26	2.08
	LUMO+2	−1.032	1.96	10.56	84.08	3.61
	LUMO+3	−1.014	3.23	10.98	84.12	1.79
	LUMO+4	−0.182	17.49	66.71	5.16	10.92

Table S4. Summary of the results of TD-DFT calculations on complex **Ir1** (assignment is provided for MO contributions＞10 %)

**No.**	**Energy (cm^{-1})**	**Wavelength (nm)**	***f***	**MOs1**	**MOs2**	**MOs3**	**MOs4**
**1**	23266	430	0.0639	HOMO→LUMO (97 %)			
**2**	28060	356	0.0135	HOMO→L+2 (97 %)			
**3**	28784	347	0.0356	HOMO→L+3 (55 %)	H−1→L+1 (31 %)	H−1→LUMO (11 %)	
**4**	29162	343	0.0543	HOMO→L+3 (42 %)	H−1→L+1 (32 %)	H−1→LUMO (20 %)	
**5**	31433	318	0.0621	H−3→L+1 (27 %)	H−2→L+1 (26 %)	H−2→LUMO (24 %)	H−3→LUMO (12 %)
**6**	32058	312	0.0144	H−2→LUMO (40 %)	H−3→LUMO (−24 %)	H−1→L+2 (20 %)	
**7**	32531	307	0.0804	H−2→L+1 (38 %)	H−3→L+1 (35 %)	H−3→LUMO (11 %)	
**8**	33130	302	0.0164	H−1→L+2 (66 %)			
**9**	33629	297	0.0225	H−1→L+3 (67 %)	H−4→LUMO (16 %)		
**10**	34344	291	0.061	H−2→L+2 (11 %)	H−4→LUMO (25 %)	H−5→LUMO (15 %),	
**11**	34889	287	0.0382	H−5→L+1 (48 %)	H−4→LUMO (29 %)		
**12**	35223	284	0.166	H−5→L+1 (30 %)	H−4→L+1 (20 %)	H−1→L+3 (11 %)	HOMO→L+4 (10 %)
**13**	36453	274	0.0832	HOMO→L+4 (40 %)	H−2→L+3 (14 %),	H−3→L+2 (12 %)	
**14**	36881	271	0.0767	H−2→L+2 (39 %)	H−3→L+2 (20 %),	HOMO→L+4 (11 %)	
**15**	37317	268	0.1275	H−3→L+3 (59 %)			
**16**	37776	265	0.3217	H−2→L+3 (57 %)			
**17**	38064	263	0.0209	HOMO→L+9 (32 %)	HOMO→L+5 (16 %)	HOMO→L+7 (17 %)	
**18**	39019	256	0.0313	H−4→L+2 (44 %)			
**19**	39056	256	0.0106	H−5→L+2 (75 %)	H−4→L+2 (13 %)		
**20**	39306	254	0.0129	H−7→L+1 (72 %)			

Table S5. Summary of the results of TD-DFT calculations on complex **Ir2** (assignment is provided for MO contributions＞10 %)

**No.**	**Energy (cm^{-1})**	**Wavelength (nm)**	***f***	**MOs1**	**MOs2**	**MOs3**	**MOs4**
**1**	24406	410	0.0632	HOMO→LUMO (97 %)			
**2**	29049	344	0.0138	HOMO→L+2 (95 %)			
**3**	29560	338	0.0691	H−1→L+1 (52 %)	H−1→LUMO (25 %)	HOMO→L+3 (15 %)	
**4**	30059	333	0.0150	HOMO→L+3 (82 %)			
**5**	31215	320	0.0129	H−3→LUMO (26 %)	H−2→LUMO (23 %)	H−4→LUMO (16 %)	H−3→L+1 (13 %)
**6**	31979	313	0.0563	H−2→L+1 (35 %)	H−2→LUMO (31 %)	H−3→L+1 (12 %)	H−4→L+1 (10 %)
**7**	32391	309	0.0349	H−2→LUMO (25 %)	H−3→LUMO (22 %)	H−2→L+1 (16 %)	
**8**	32994	303	0.0944	H−3→L+1 (31 %),	H−2→L+1 (22 %)	H−3→LUMO (16 %)	H−4→L+1 (14 %),
**9**	33899	295	0.0256	H−4→LUMO (39 %)	H−1→L+3 (35 %)	H−3→LUMO (15 %)	
**10**	34342	291	0.0535	H−4→L+1 (35 %)	H−3→L+1 (17 %)	H−1→L+3 (16 %)	
**11**	34830	287	0.0641	H−5→LUMO (37 %)	H−1→L+3 (14 %)	H−3→L+1 (10 %)	
**12**	35150	284	0.1061	H−5→LUMO (49 %)	H−5→L+1 (−12 %)		
**13**	35355	283	0.0604	H−5→L+1 (71 %)			
**14**	36056	277	0.0409	H−2→L+2 (43 %)	H−3→L+2 (12 %)	H−6→LUMO (10 %)	
**15**	36417	275	0.1176	HOMO→L+4 (56 %)	H−3→L+2 (11 %),		
**16**	36924	271	0.1000	H−6→L+1 (46 %)	H−2→L+3 (14 %)	H−3→L+2 (15 %)	
**17**	37844	264	0.1389	H−3→L+3 (42 %)	H−4→L+3 (19 %)		
**18**	37944	264	0.1769	H−2→L+3 (43 %)	H−6→L+1 (18 %)	H−3→L+3 (13 %)	
**19**	38287	261	0.0192	HOMO→L+7 (27 %)	H−1→L+7 (11 %)	H−3→L+2 (10 %)	
**20**	38583	259	0.0887	H−4→L+2 (52 %)	H−3→L+2 (17 %)		

Table S6. Summary of the results of TD-DFT calculations on complex **Ir3** (assignment is provided for MO contributions＞10 %)

No.	Energy (cm^{-1})	Wavelength (nm)	*f*	MOs1	MOs2	MOs3	MOs4
**1**	25583	391	0.0536	HOMO→L+1 (84 %)	HOMO→LUMO (14 %)		
**2**	30227	331	0.0842	H−1→L+1 (77 %)	H−1→LUMO (11 %)		
**3**	31566	317	0.0172	HOMO→L+2 (91 %)			
**4**	32441	308	0.1099	H−2→LUMO (57 %)	H−2→L+1 (15 %)		
**5**	33183	301	0.1304	H−3→L+1 (53 %)	H−1→L+2 (13 %)	H−3→LUMO (10 %)	
**6**	33348	300	0.0149	H−3→LUMO (59 %)	H−3→L+1 (13 %)		
**7**	33882	295	0.0363	H−4→LUMO (26 %)	H−4→L+1 (23 %)	H−2→L+1 (14 %)	
**8**	34015	294	0.1036	H−4→LUMO (26 %)	H−4→L+1 (24 %)	H−3→LUMO (11 %)	
**9**	34809	287	0.0147	H−5→LUMO (29 %)	H−6→LUMO (16 %)	H−4→LUMO (14 %)	H−4→L+1 (10 %)
**10**	35783	279	0.0470	H−1→L+2 (35 %)	HOMO→L+4 (21 %)	H−6→L+1 (10 %)	
**11**	36020	278	0.0136	H−6→L+1 (43 %),	HOMO→L+4 (18 %)	H−5→L+1 (16 %)	
**12**	36479	274	0.1804	H−1→L+2 (29 %)	HOMO→L+4 (14 %)	HOMO→L+6 (11 %)	H−6→L+1 (11 %)
**13**	37357	268	0.0105	HOMO→L+7 (30 %)	HOMO→L+5 (24 %)		
**14**	37760	265	0.0348	H−2→L+2 (57 %)			
**15**	38037	263	0.0100	HOMO→L+5 (−16 %)	HOMO→L+7 (16 %)	H−1→L+7 (13 %)	H−2→L+2 (−10 %)
**16**	38297	261	0.2198	H−2→L+3 (65 %)			
**17**	38716	258	0.0364	H−3→L+2 (65 %)			
**18**	39817	251	0.0240	H−4→L+3 (64 %)			
